# Prophylactic effects of biogenic selenium nanoparticles on acute toxoplasmosis: An in vivo study

**DOI:** 10.1016/j.amsu.2020.04.010

**Published:** 2020-04-29

**Authors:** Mojtaba Shakibaie, Fatemeh Ezzatkhah, Esraa Gabal, Ebrahim Badparva, Sareh Jahanbakhsh, Hossein Mahmoudvand

**Affiliations:** a Pharmaceutical Sciences and Cosmetic Products Research Center, Kerman University of Medical Sciences, Kerman, Iran; b Department of Laboratory Sciences, Sirjan School of Medical Sciences, Sirjan, Iran; c Agricultural Science and Resource Management in the Tropics and Subtropics, Bonn University, Germany; d Razi Herbal Medicines Research Center, Lorestan University of Medical Sciences, Khorramabad, Iran; e Student Research Committee, Lorestan University of Medical Sciences, Khorramabad, Iran

**Keywords:** Prophylactic, Selenium, *Toxoplasma gondii*, Treatment, Toxicity

## Abstract

**Background:**

In this investigation, the in vivo efficacy and safety of biogenic selenium nanoparticles (SeNPs) are assessed against acute toxoplasmosis caused by *Toxoplasma gondii* (Sarcocystidae) in the mice.

**Methods:**

Male NMRI mice were orally treated with normal saline (control group) and SeNPs at the doses of 5 and 10 mg/kg once a day for 14 days*.* On the 15th day, the mice were infected with 10^4^ tachyzoites of *T. gondii* RH strain by the intraperitoneal route. The mortality rate and parasite load were determined in the infected mice. The mRNA levels of IFN-γ, IL10, IL12, and inducible nitric oxide synthase were also examined in the infected mice by quantitative real-time PCR.

**Results:**

The rate of mortality in the infected mice receiving SeNPs at the doses of 5 and 10 mg/kg compared with the mice in the control group was 100% on the 9 and 10 days after the administration. The mean number of tachyzoites in the infected mice receiving SeNPs was significantly lower than that in the control group. No significant difference (*p* > 0.05) was found in the biochemical parameters between the mice treated with SeNPs and the mice in the control group. The results revealed that mRNA levels significantly improved in the infected mice treated with SeNPs compared with those in the control group.

**Conclusion:**

Findings of the present investigation showed the considerable efficacy of SeNPs with no important toxicity for curing acute toxoplasmosis in the mice model. However, further studies are needed to clarify the accurate anti-Toxoplasma mechanisms of SeNPs.

## Highlights

• We evaluated the efficacy of Biogenic Selenium Nanoparticles (SeNPs) against acute toxoplasmosis.
• Mice were orally treated with SeNPs at the doses of 5 and 10 mg/kg once a day for 14 days.
• The average number of tachyzoites and mortality rate was significantly lower in treated mice.
• No significant difference (*p* > 0.05) observed in the biochemical parameters between the mice treated.
• The mRNA levels of meaningfully improved in infected mice treated with SeNPs.

## 1 Introduction

*Toxoplasma gondii* as a universal intracellular parasite which is observed all over the world infects a broad spectrum of animals and about 30% of humans (1(. At present, the three main ways of infection by this parasite, which are almost proven, are as follows: (i) digestion of raw or undercooked meat contaminated with tissue cysts, (ii) eating of sporulated oocysts with food or (iii) congenitally from mother to fetus during pregnancy)^2^, ^3^(. In relation to the clinical signs of the *T. gondii* infections (toxoplasmosis), there are various forms of this disease, from asymptomatic forms to dangerous and, even deadly, ones)^4^, ^5^(. Nowadays, the best medication used to treat toxoplasmosis is the concomitant use of pyrimethamine and sulfadiazine; however, studies in recent years have suggested that the side effects of these drugs such as osteoporosis, sepsis, and teratogenic properties, especially in immuno compromised individuals, should not be neglected [[6], [7], [8]]. Considering the explanations given above, it seems that finding a new drug with the same and even higher effectiveness as well as less toxicity would be the intellectual concern for researchers in the world.

Nowadays, it has been proven that selenium (Se) is one of the most important and vital elements for humans and animals, the deficiency of which causes irreparable diseases and injuries such as immune impairment [9]. This element exists in a range of functional proteins that play vital roles including anti-cancer effects and strengthen the immune system against infectious agents [10,11]. Since nanoparticles (NPs) have a high surface-to-volume ratio, they have extensive biological activities [12]. Several investigations have demonstrated that NPs, especially Se NPs, can considerably prevent the growth of some microbial pathogenic strains such as *Staphylococcus aureus*, *Escherichia coli*, and *Leishmania* spp [13,14].

Although the main antimicrobial mechanisms of these nanoparticles are still unclear, a number of investigations have reported that some forms of selenium can show the potent antimicrobial effects through reaction with membrane peroxidases and, subsequently, produce oxygen-free radicals [15]. On the other hand, some studies have exhibited that biogenic Se NPs by activating apoptosis in the *Leishmania major* promastigotes show their antimicrobial mechanisms [16,17].

Since there is no documentary study on the efficacy of biogenic Se NPs for acute toxoplasmosis, the present study was designed to evaluate the efficacy of biogenic Se NPs against acute toxoplasmosis in the mice model.

### 1.1 Materials and methods

#### 1.1.1 Biosynthesis and characterization of the Se NPs

In the present investigation, biosynthesis of biogenic Se NPs was carried out according to the method performed by Shakibaei et al. [9]. After the extraction and purification of the Se NPs, the electron microscopy of SeNPs was conducted by carbon-coated copper transmission electron microscope grids and dried under an IR lamp. Moreover, the crystalline structure of the Se NPs was assessed using the X-ray diffraction (XRD) as previously explained [9].

### 1.2 Animals

Forty-eight male NMRI mice aged 40–45 days and weighting from 20 to 25 g were obtained from Pasteur Institute, Tehran, Iran, to induce the acute toxoplasmosis and safety of Se NPs. Mice were kept in a colony room with 12 h of light and 12 h of darkness at the room temperature.

### 1.3 Ethical statement

The protocol of this survey was permitted by Ethics Committee of Kerman University of Medical Science (Permit Number: 93/110). The work was also carried out in line with the ARRIVE Guidelines for Reporting Animal Research [10].

### 1.4 Parasite

The *T. gondii* virulent RH strain was prepared by Department of Parasitology and Mycology, Kerman University of Medical Sciences, Kerman, Iran. Tachyzoites of *T. gondii* RH strain (1 × 10^4^) was inoculated intraperitoneally (IP) to the mice in order to establish an animal model of acute toxoplasmosis.

### 1.5 Experimental design

**Fig. 1:**
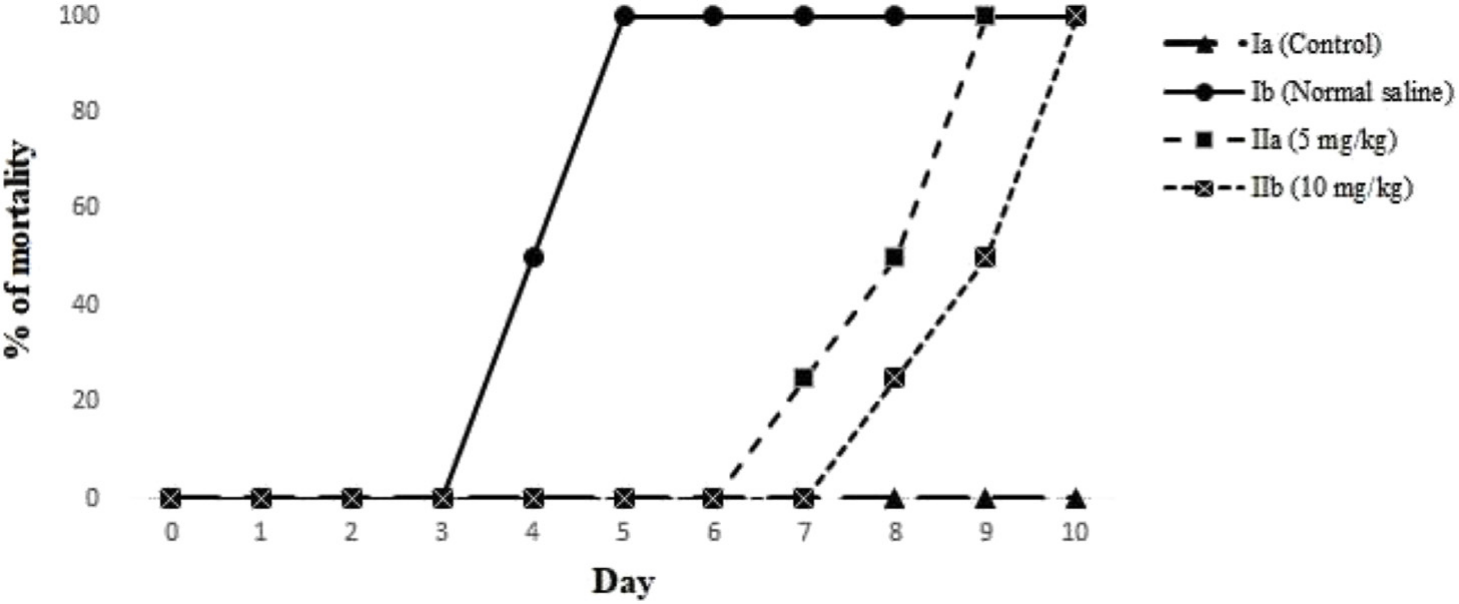
Effect of oral administration of SeNPs on mortality rate of mice with acute toxoplasmosis infected with *T. gondii* RH strain.

Fig. 1 shows the experimental design of the present study. After treatment of the mice with SeNPs at the dose of 5 and 10 mg/kg once a day for 14 days, the mice were infected with 1 × 10^4^ tachyzoite of *T. gondii* RH strain IP. Then, the following parameters were performed to evaluate the efficacy of oral SeNPs against acute toxoplasmosis.

### 1.6 Mortality rate (MR)

The following formula was applied to calculate the MR:

$$\text{MR}\;=\;\frac{\text{Number of dead mice}}{\text{Number of mice at the beginning of the experiment}}\times 100$$

### 1.7 Parasite load

After the aspiration of the peritoneal fluid of each of the infected mice, the mean number of the *T. gondii* tachyzoites was recorded under light microscope using the 10 X high power field [11].

### 1.8 Evaluating safety of SeNPs on biochemical parameters

In order to evaluate the toxicity effects of SeNPs, the mice in the subgroups 1c1 and 1c2 compared with those in the control group Ia were fasted overnight. Animals were anesthetized using ketamine (100 mg/kg)-xylazine (10 mg/kg) and, after opening thoracic cavity, total blood was collected from the heart of the mice. For assessment of the serum biochemical factors, the rest of the collected blood samples was put into tubes without any anticoagulant. Then, the serum was collected using centrifugation at 5000*g* for 5 min. In the next step, some of the main biochemical factors including aspartate aminotransferase (AST), alanine aminotransferase (ALT), alkaline phosphatase (ALP), creatinine (Cr), blood urea nitrogen (BUN), and bilirubin (direct and total) were measured using the Roche Diagnostics kits (Mannheim, Germany) [12].

### 1.9 Analysis of mRNA expression by real-time PCR

Since selenium affects all components of the immune system, particularly cellular immunity, the mRNA levels of IFN-γ, IL10, and IL12 as well as inducible nitric oxide synthase (iNOs) as the main effective mechanisms for control and resistance to *T. gondii* during infection were examined in the infected mice treated with SeNPs by quantitative real-time PCR.

To extract the total RNAs from the blood samples, commercial RNeasy kits (Qiagen, Hilden, Germany) were employed. After the extraction, all the obtained RNAs were reverse transcribed by means of RT premix kit (Qiagen, Hilden, Germany) based on the manufacture's procedure. Then, complementary DNA (cDNA) was used not only for the conventional PCR, but also for real-time PCR.

Real-time PCR was carried out via the iQ5 real-time PCR detection system (Bio-Rad, Hercules, CA), whereas the SYBR green was applied to determine the products [13]. Conditions of reactions were established according to the method described elsewhere. Finally, the obtained data was analyzed by means of iQ™5 Optical System software (Bio-Rad). Table 1 indicates the primer sequences used for IFN-γ TNF-α, IL-12, iNO, and β-actin (house-keeping gene).

**Table 1:** Sequences of primers used for real-time PCR.

Amplicon	Primers	Sequence (5′–3′)
IL12	F	ACGACATTCGTCAACTGCAA
	R	TAAATTGGCACCCTGTAGGC
IFN-γ	F	GATCGTGTCGTCACCAGAAAGG
	R	TGCCTGGTAACGAGTTGTCC
NO	F	CGTGAAAGGATCCAGAAAGG
	R	TCCACGATGCCTGGTAGTTG
TNF-α	F	ATTTCTCACGCCAGGATTTG
	R	GATCGGCAAAGGTTAGGTCA

### 1.10 Statistical analysis

Analysis of the data was performed by SPSS statistical software (ver. 22.0). To compare the differences between the experimental groups, one-way ANOVA with Tukey's post-hoc test was applied. *p* < 0.05 was also measured as statistically significant.

## 2 Results

### 2.1 Estimation of the mortality rate

Fig. 1 demonstrates the mortality rate of the infected mice after treatment with SeNPs at doses of 5 and 10 mg/kg for two weeks. The results indicated that the rate of mortality in the infected mice receiving SeNPs at the doses of 5 and 10 mg/kg compared with the mice in control group was 100% on 9 and 10 days after the administration. Obtained findings exhibited that there was a significant difference (P < 0.05) in survival rate among the mice receiving SeNPs with the mice in the control group.

### 2.2 Parasite load

The findings also demonstrated that the mean number of tachyzoites in the infected mice receiving SeNPs at the doses of 5 and 10 mg/kg was 127 × 10^4^ and 56 × 10^4^ and significantly lower than those in the control group (288 × 10^4^ tachyzoites).

### 2.3 Safety of SeNPs on biochemical parameters

To examine the safety of SeNPs, some main biochemical parameters were evaluated on the mice in the subgroups 1c1 and 1c2 in comparison with those in the control group 1a after two weeks of SeNPs administration. There was no mortality in the mice in the subgroups 1c1 and 1c2 after two weeks. The findings of the biochemical parameters are presented in Table 2. These results revealed no significant difference (*p* > 0.05) in the biochemical parameters between the mice in the subgroups 1c1 and 1c2 compared with those in the control group Ia.

**Table 2:** Clinical biochemistry parameters in serum of tested mice.

Clinical biochemistry parameters	BUN(mg/dL)	Cr (mg/dL)	AST (U/L)	ALT (U/L)	ALP (U/L)	TB
Control	31.4	0.24	130.6	38.7	133.3	0.15
Se NPs (5 mg/kg)	33.5	0.29	141.8	44.2	137.2	0.16
Se NPs (10 mg/kg)	36.6	0.31	148.3	47.7	140.1	0.18

BUN, Blood urea nitrogen; Cr, creatinine; ALT, alanine aminotransferase; ALP, alkaline phosphatase; AST, aspartate aminotransferase; TB, total bilirubin.

### 2.4 Analysis of cytokine mRNA expression by real-time PCR

**Fig. 2:**
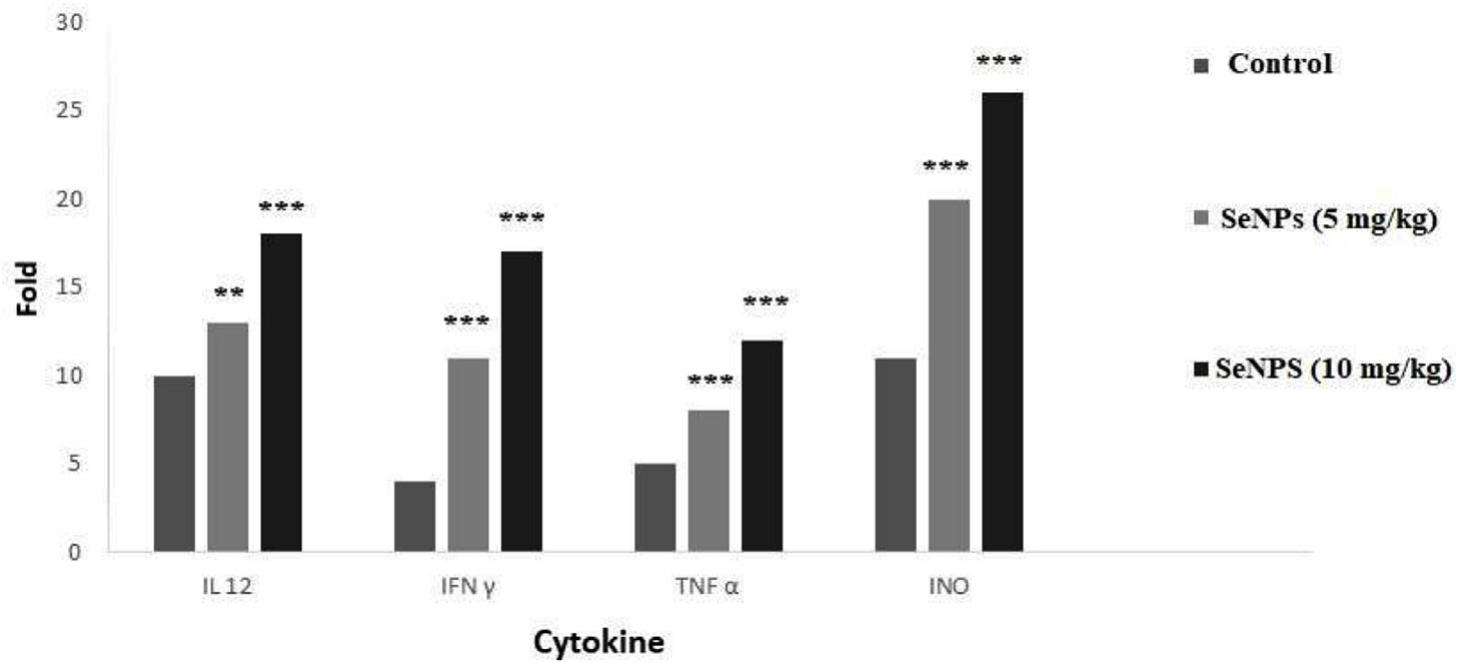
The mRNA levels of IFN-γ, TNF-α, IL-10, IL-12, as well as iNO in mice. **p < 0.01; ***p < 0.001 statistically significant.

The mRNA levels of IFN-γ, TNF-α, IL-12, as well as iNO were assessed in the infected mice using real-time PCR. As demonstrates in Fig. 2, the results revealed that mRNA levels of IFN-γ (P < 0.001), TNF-α (P < 0.001), IL-12 (P < 0.05), and iNOs (P < 0.05) significantly improved in the infected mice treated with SeNPs compared with those in the control group.

## 3 Discussion

Currently, the main treatment for toxoplasmosis is the use of the combination of pyrimethamine and sulfadiazine; however, this combination therapy has some limitations because of possessing dangerous side effects, mainly in the patients with immune system disorders such as AIDS patients [2,3]. Therefore, it seems necessary to search for an alternative agent with higher efficacy as well as less side effects.

The obtained findings revealed that the rate of mortality in the infected mice receiving SeNPs at the doses of 5 and 10 mg/kg compared with the mice in the control group was 100% on days 9 and 10 after the administration. Furthermore, the mean number of tachyzoites was considerably reduced for the infected mice treated with SeNPs at the doses 5 and 10 mg/kg.

Today, selenium compounds have shown a wide range of therapeutic applications such as non-carcinogenic, antioxidant, and antimicrobial [[6], [7], [8]]. In recent years, studies have demonstrated that nanoparticles can be applied for various therapeutic purposes, since these particles have small nanoscale sizes as well as great surface-to-volume percentages, which could produce more active areas for cooperating with biological molecules such as microbes.

In toxoplasmosis, both innate and adaptive immune responses (humoral and cellular immunity), are required to control this infection in humans [14]. Reviews have reported that selenium can motivate the immune responses; moreover, Yazdi et al. (2015) demonstrated that, by sandwich ELISA, levels of cellular immunomodulatory cytokines such as granzyme B, IL-12, IFN-γ, and IL-2 considerably improved (*P* < 0.05) in the mice treated with both SeNPs [15,16]. Similarly, it was found that the expression levels of IFN-γ TNF-α, IL-12, IL-10, as well as iNO were considerably increased in the infected mice treated with SeNPs compared with the untreated BALB/c mice. These results indicated that the increased survival time in the infected mice after the oral administration of SeNPs might be due to the strengthening of the immune system, especially the cellular immunity of the animal, which caused resistance to the infection.

Several investigations have reported the antimicrobial activity of SeNPs; for example, Beheshti et al. (2013) demonstrated that Se NPs as a new antileishmanial drug might be used to treat the cutaneous leishmaniasis in mice [17]. In another work, Se NPs, particularly in combination with meglumine antimoniate, was reported to considerably inhibit the promastigote and amastigote stages of sensitive and glucantime resistance Leishmania tropica in the in vitro conditions. Mahmoudvand et al. [18] demonstrated that Se NPs could be described as a novel protoscolicidal agent during hydatid cyst surgery. Other studies conducted by Tran et al. [19] and Yang et al. [20] have shown that Se NPs have potent efficacy to prevent the growth of *S. aureus* and *E. coli*.

Nowadays, in order to investigate the sub-acute toxicity of some pharmacological agents in animal models, researchers employ the evaluation of the liver and renal enzyme activities such as ALT, AST, ALP, bilirubin (total, direct), Cr, and BUN as well as hematological parameters. These results reveal no significant difference (*p* > 0.05) in the biochemical and hematological parameters between the mice in the subgroups 1c1 and 1c2 compared with those in the control group Ia. These results consistent with the toxicity classification indicate that SeNPs have no significant toxicity against the male BALB/c mice [21].

## 4 Conclusion

The findings of the present investigation showed the considerable efficacy of SeNPs with no important toxicity to cure the acute toxoplasmosis in the mice model. However, further studies are needed to clarify the accurate anti-*Toxoplasma* mechanisms of SeNPs.

## Availability of data and materials

All data generated or analyzed during this study are included in this published article.

## Funding

None.

Provenance and peer review.

Not commissioned, externally peer reviewed.

## Declaration of competing interest

The authors declare that they have no competing interests.
